# From Burden to Breakthrough: The Impact of Mass Drug Campaigns on Microfilaria Trends Based on Evidence From Sentinel Site Surveillance in Rural Nagpur, Maharashtra, India, Over Two Decades

**DOI:** 10.7759/cureus.90751

**Published:** 2025-08-22

**Authors:** Smita V Mohod, Dilip S Gedam, Prashant Meshram, Vasundhari Potsangbam

**Affiliations:** 1 Department of Microbiology, Indira Gandhi Government Medical College and Hospital, Nagpur, IND

**Keywords:** diethylcarbamazine citrate, lymphatic filariasis, mass drug administration, microfilaria rate, national vector borne disease control programme

## Abstract

Background

Lymphatic filariasis (LF), often referred to as elephantiasis, is a neglected tropical disease that severely impacts health, causing chronic conditions such as lymphoedema and hydrocele. In India, it affects millions, leading to disability and social stigma, making elimination a priority. The National Vector Borne Disease Control Programme (NVBDCP) has been key in managing LF through mass drug administration (MDA), which traditionally used single-drug or two-drug regimens. The introduction of the triple-drug therapy in 2018, particularly in Nagpur, marks a shift to enhance effectiveness, aiming for faster transmission interruption. This study aims to assess the demographic and temporal distribution of LF and to evaluate the effectiveness of the triple-drug MDA in reducing key transmission indicators in Nagpur.

Materials and methods

This was a retrospective analysis of all eligible records (n = 36,080 individuals) available from the district LF sentinel-surveillance system between January 2004 and December 2024. Data were collected from four sentinel primary health centres (PHCs) (fixed spots): Titur, Bhiwapur, Gumthala, and Umred, covering the villages of Sili, Salebhatti, Bhowari, and Rajebabaward. From 2021, Takalghat and Borkhedi PHCs (villages: Turakmari and Ruikhiri) were added to monitor MDA coverage and microfilaria (MF) rates. The p-value of the MF rate in each year was calculated using a chi-square test analysis, which was performed with IBM SPSS Statistics for Windows, Version 24 (Released 2016; IBM Corp., Armonk, New York).

Results

A total of 36,080 individuals were surveyed, comprising 19,168 (53%) males and 16,912 (47%) females, with a female-to-male ratio of 0.88:1. The mean age of the study population was 30.67 ± 13.71 years (95% CI: 30.53-30.81 years), with a median age of 30.5 years. MF was detected in 441 individuals (1.22%), with a higher prevalence among males (285, 64.62%) compared to females (156, 35.37%). The highest MF positivity was recorded in the 6-15 age group, followed by the 16-25 age group, indicating greater vulnerability among younger individuals. Over the two-decade study period, MF cases declined substantially from 102 cases in 2004 to zero by 2022, demonstrating the sustained impact of MDA. Overall, the prevalence dropped from 4.91% in 2004 to 0% by 2024, reflecting the success of long-term filariasis elimination efforts in the Nagpur district.

Conclusion

The successful implementation of the triple-drug MDA strategy has significantly halted LF transmission in Nagpur, Maharashtra. This outcome highlights the effectiveness of the IDA (ivermectin, diethylcarbamazine, and albendazole) regimen when combined with strong public health efforts. To sustain elimination, continued community engagement and vigilant post-MDA surveillance are essential. The Nagpur experience serves as a model for other endemic regions aiming to achieve long-term control and eventual elimination of lymphatic filariasis.

## Introduction

Filariasis is a mosquito-borne parasitic disease caused by nematodes of the family *Filarioidea*, with *Wuchereria bancrofti*, *Brugia malayi*, and *Brugia timori* being the principal agents of lymphatic filariasis (LF) [1]. Transmission occurs through bites of infected mosquitoes, predominantly *Culex quinquefasciatus* in urban and semi-urban areas, *Anopheles* species in rural settings, and *Aedes* species in certain island regions [2]. After entering the human host, the larvae mature into adult worms within the lymphatic system, leading to lymphatic dysfunction of varying severity.

LF is endemic across tropical and subtropical regions, posing a significant global health challenge. More than 860 million people are at risk worldwide, with India alone accounting for approximately 40% of the global burden [3]. Within India, the disease is endemic in 20 states and union territories, where nearly 740 million people across 345 districts remain vulnerable [4].

Clinically, LF ranges from asymptomatic infection with microfilaremia still contributing to transmission to acute presentations such as adeno-lymphangitis, fever, and localized inflammation. Chronic manifestations, including lymphoedema, hydrocele, and elephantiasis, cause lifelong disability, social stigma, and economic loss [5]. The disease is recognized as the world's second leading cause of long-term disability [6].

Diagnosis has traditionally relied on the detection of microfilariae (MF) in peripheral night blood smears stained with Giemsa. More sensitive methods, such as immunochromatographic card tests (ICT) and ELISA (enzyme-linked immunosorbent assay) for circulating filarial antigen (CFA), have enabled daytime testing, while molecular methods like PCR and imaging techniques, such as ultrasound ("filarial dance sign"), further enhance diagnostic accuracy, though they remain limited in routine use [7,8].

India has committed to eliminating LF by 2027. The cornerstone strategy is mass drug administration (MDA) with a triple-drug regimen: albendazole (400 mg), ivermectin (150-200 µg/kg), and diethylcarbamazine (6 mg/kg), administered biannually, along with National Deworming Day. This triple-drug therapy (IDA) was successfully piloted in Nagpur, Maharashtra, in January 2019 [9,10]. The 2025 Phase-I MDA campaign will cover 109 districts in 13 states, reflecting India's mission-mode approach. The national MDA coverage has improved steadily, from 72.42% in 2004 to 87.25% in 2019 [11]. Sentinel surveys conducted annually in endemic districts remain essential for monitoring disease trends, evaluating programmatic impact, and identifying high-risk populations. Such evidence is crucial to guide targeted interventions and strengthen elimination efforts.

The objective of the study is to assess the distribution of LF in selected sentinel areas across rural Nagpur, with a specific emphasis on age-sex-wise patterns, and to evaluate the evidence-based effectiveness of MDA on the microfilaria rate (MF rate) in selected sentinel areas in rural Nagpur, Maharashtra, India.

## Materials and methods

Nagpur, the sub-capital of Maharashtra, India, is spread over an area of 9,892 km² with an estimated population of 46 lakh as per the 2011 census. This retrospective cross-sectional database study, spanning two decades (January 2004 to December 2024), was conducted to evaluate changes in the MF rate in the Nagpur district. The study period captures critical epidemiological shifts both before and after the introduction of the triple-drug regimen (IDA) for filariasis elimination. Since 2004, a total of 18 rounds of MDA have been completed up to 2025, excluding 2016 and 2020 (when additional MF surveys were undertaken) and 2021 (when the Transmission Assessment Survey (TAS) was conducted).

The Nagpur district comprises 46 primary health centres (PHCs). In line with the National Vector Borne Disease Control Programme (NVBDCP) guidelines, four sentinel PHCs (Titur, Bhiwapur, Gumthala, and Umred) were purposively selected based on historical endemicity, programmatic importance (such as low MDA compliance or high migration), and ecological/geographical representation. Once chosen, these fixed sites were retained across survey rounds to enable long-term trend analysis. From each selected sentinel PHC, one representative village (Sili, Salebhatti, Bhowari, and Rajebabaward, respectively) was monitored. Within these villages, night blood smears were systematically collected from a fixed proportion of the general population to ensure comparability and not just suspected cases across survey years.

From 2021 onwards, two additional PHCs (Takalghat and Borkhedi, covering villages Turakmari and Ruikhiri, respectively, as fixed spots) were included to assess MDA coverage under the NVBDCP and to monitor the MF rate through corresponding sentinel survey data. The study emphasized both demographic and temporal distribution patterns of LF, with the MF rate calculated based on the presence of MF in peripheral blood smear (PBS) examinations. The insights derived from this study aim to inform and enhance the strategic planning and implementation of public health interventions in the region. Data collection, compilation, and statistical analysis were conducted using IBM SPSS Statistics for Windows, Version 24 (Released 2016; IBM Corp., Armonk, New York) by the Department of Microbiology at Tertiary Care Hospital and Teaching Institute, Nagpur, Maharashtra, India.

The study population consisted of residents from designated sentinel sites, selected for longitudinal surveillance of *Wuchereria bancrofti* infection under the NVBDCP. As part of routine programmatic activities, nocturnal peripheral blood samples were collected and examined microscopically for the presence of MF. These diagnostic procedures were implemented across identified healthcare facilities within the study regions over a 20-year period, from January 2004 to December 2024. Microscopic examination of night blood smears remains the gold standard for diagnosing LF in endemic settings, providing direct parasitological confirmation of active infection.

As per the World Health Organization (WHO) guidelines [6], a clinical case of lymphatic filariasis is defined as an individual living in an LF-endemic region who shows signs of hydrocoele or lymphoedema, with other possible causes ruled out. The laboratory confirmation of the disease can be established by detecting MF in peripheral blood samples, identifying CFAs, or finding histological evidence through a tissue biopsy. Classification of cases includes the following: suspected cases are not defined in the context of LF; probable cases meet the clinical criteria without lab confirmation; and confirmed cases are those with definitive laboratory evidence, regardless of the presence of clinical symptoms.

Peripheral blood specimens, collected at nighttime (10 pm to 2 am), were obtained from all individuals (> 5 years of age group) residing within the study area through both passive surveillance (health facility-based) and active surveillance (community-based household visits). The identification and differentiation of filarial parasites were performed via microscopic examination of Giemsa-stained PBSs (considered as a gold standard), in adherence to the diagnostic standards outlined under the NVBDCP guidelines. Smear screening and positivity were performed by trained healthcare workers and laboratory technicians for quality assurance.

Data from MF sentinel surveys spanning two decades were included in this study, encompassing the general population (age group >5 years to < 85 years) residing in fixed sentinel spots across rural Nagpur. Only data officially recorded by the respective PHCs and documented within the NVBDCP framework were considered, while duplicate entries were excluded.

Ethical approval was obtained from the Institutional Ethics Committee of Indira Gandhi Government Medical College and Hospital (reference ID: IGGMC/Pharmacology/IEC/149/2025, dated 10/06/25).

The dataset comprised the total number of peripheral blood samples collected from individuals presenting at each PHC between January 2004 and December 2024, obtained through both active (household-based) and passive (facility-based) surveillance under the NVBDCP framework. It further included the number of Giemsa-stained PBSs that tested positive for MF. Laboratory-confirmed cases of LF were stratified based on demographic characteristics (such as age and sex) and temporal distribution over the study period. Parasitological indices were computed to assess disease burden using the standard formula:

$$ \text{Microfilaria Rate (%)} = \left( \frac{\text{Number of individuals positive for microfilariae}}{\text{Total number of individuals examined}} \right) \times 100 $$ The p-value of the MF rate in each year was calculated using the chi-square test.

## Results

The total number of samples surveyed from 2004 to 2024 was 36,080. The survey was not conducted during the years 2016, 2020, and 2021. Of the total 36,080 samples, 19,168 (53.1%) (95% CI = 52.61%-53.65%) were males and 16,912 (46.9%) (95% CI = 46.35%-47.39%) were females, yielding a female-to-male (F:M) ratio of 0.88:1. The mean age of the study population was 30.67 ± 13.71 years (95% CI = 30.53-30.81 years), with a median age of 30.5 years (Table 1).

**Table 1:** Demographic characteristics of the study participants from 2004 to 2024

Characteristics	Values	95% Confidence Interval
Total number of surveyed population (N)	36,080	-
Gender, n (%)		
Male	19,168 (53.13%)	52.61%–53.65%
Female	16,912 (46.87%)	46.35%–47.39%
F:M ratio	0.88:1	-
Age (years)		
Mean age ± SD	30.67 ± 13.71 years	30.53–30.81 years
Median age	30.5 years	-

**Figure 1:**
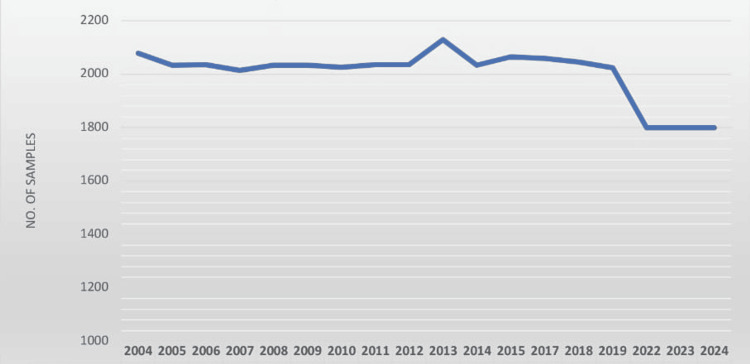
Blood samples collected from 2004 to 2024 in the study population (N=36,080)

Between 2004 and 2024, no surveys were conducted in the years 2016, 2020 (due to the additional MF survey), and 2021 (due to the TAS). Sample numbers remained consistent (~2000-2100/year) until 2020, with peaks in 2013 and 2019. From 2019 onward, a decline was observed, with approximately 1800 samples collected annually, indicating reduced surveillance activity or operational shifts (Figure 1).

**Figure 2:**
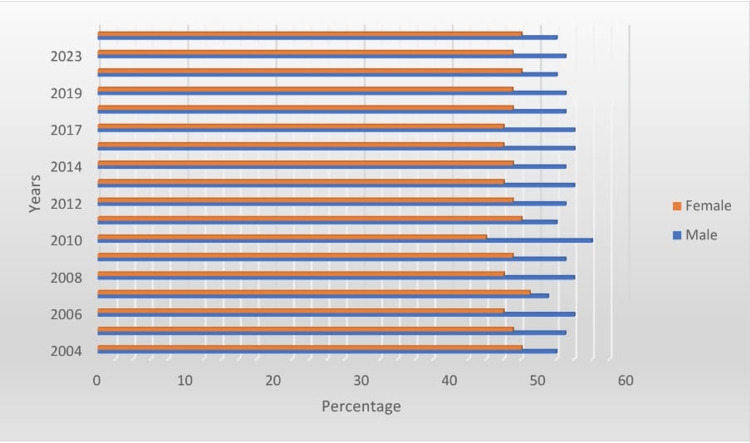
Gender-wise percentage distribution of the total surveyed population in each year from 2004 to 2024 (N=36,080)

Figure 2 depicts the gender-wise percentage distribution of the total surveyed population from 2004 to 2024, showing a consistent pattern in the representation of males and females across the study period. The data indicate that the percentage of female participants remained relatively stable, ranging approximately between 45% and 50% annually. In contrast, the male population consistently accounted for the remaining proportion, fluctuating between 50% and 55% over the same timeframe. This near-equitable distribution suggests a balanced gender representation in the surveyed population, with no significant shifts observed across the 20-year span from 2004 to 2024. These findings provide a foundational demographic context for subsequent analyses of filariasis prevalence and associated factors within the study cohort.

**Figure 3:**
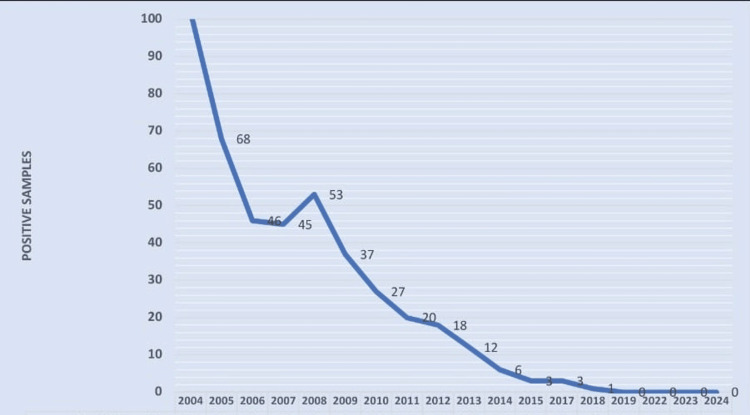
Microfilaria-positive cases in peripheral blood smear from 2004 to 2024 in the study population (n=441)

Figure 3 shows that, out of 36,080 blood samples screened from 2004 to 2024, a total of 441 were positive for MF. The number of positive cases declined sharply from 102 in 2004 to zero by 2022. A consistent downward trend was observed, with minor fluctuations between 2008 and 2012. No MF-positive cases were detected in the past three years (2022-2024), indicating a possible interruption in transmission.

Table 2 revealed a significant decline in MF-positive cases from 2004 (n = 102) to 2024 (n = 0), with an initial higher prevalence among males (e.g., 72 vs. 30 in 2004). Chi-square tests indicated significant gender differences in 2004 (p < 0.0001) and 2005 (p = 0.002), but not in subsequent years (p > 0.05), reflecting a diminishing trend. The MF rate decreased from 4.91% in 2004 to 0% by 2024, suggesting a potential eradication of MF positivity in the population.

**Table 2:** Microfilaria rate from 2004 to 2024 in the study population (N=36,080)

Year	MF-Positive Males (n=285, 64.62%)	MF-Positive Females (n=156, 35.37%)	Total MF Positive (n=441, 1.22%)	MF Rate (%)	Chi-square Value (x^2^)	p-value
2004	72	30	102	4.91	14.28	<0.0001
2005	48	20	68	3.34	8.76	0.002
2006	29	17	46	2.26	1.19	0.13
2007	26	19	45	2.23	0.84	0.17
2008	31	22	53	2.61	0.45	0.25
2009	21	16	37	1.82	0.21	0.32
2010	17	10	27	1.33	0.53	0.23
2011	13	7	20	0.98	1.36	0.12
2012	11	7	18	0.88	0.48	0.24
2013	8	4	12	0.56	0.78	0.19
2014	4	2	6	0.3	0.45	0.25
2015	2	1	3	0.15	0.44	0.02
2017	2	1	3	0.14	0.19	0.44
2018	1	0	1	0.04	0.003	0.47
2019	0	0	0	0	-	-
2022	0	0	0	0	-	-
2023	0	0	0	0	-	-
2024	0	0	0	0	-	-

Table 3 shows the age-wise MF rate overall. The MF rate is highest in the youngest age group (6-15 years) and decreases progressively with age. This pattern aligns with the observation that the MF rate is maximum in the 6-15 years group, followed by the 16-25 years group.

**Table 3:** Microfilaria rate according to age in the study population MF: microfilaria

Age Group (Years)	Blood Sample Collected	MF Positive	MF Rate (%)
6–15	6234	112	1.79
16–25	8231	129	1.57
26–35	7120	89	1.25
36–45	7310	68	0.93
>45	7185	43	0.60

## Discussion

LF not only causes physical disability but also exerts significant psychosocial impacts, particularly in marriage-related social dynamics. Individuals with visible symptoms like lymphedema and hydrocele frequently encounter social stigma, which is more severe in women. Cultural beliefs often link these deformities with impurity or hereditary disease, creating barriers to marriage and leading to emotional distress, social rejection, and long-term socio-economic disadvantages [12]. This psychosocial burden reinforces poverty and marginalization, indicating the need for comprehensive strategies beyond medical treatment.

A total of 36,080 individuals were surveyed, comprising 19,168 males (53%) (95% CI = 52.61%-53.64%) and 16,912 females (47%) (95% CI = 46.36%-47.39%, resulting in a female-to-male ratio of 0.88:1 (Table 1, Figures 1, 2). This confidence interval for the gender distribution ensures that males showed a higher degree of confidence than females, indicating that the proportion of males is significantly higher than the proportion of females.

Out of the total population surveyed, 441 individuals tested positive for MF, which represents 1.22% of the population (Table 2). This low infection rate suggests a declining burden of disease in the surveyed area, reflecting the impact of long-term control measures. However, even a small percentage may signify ongoing transmission risks if not addressed with vigilant surveillance.

The sharp decline from 102 in 2004 to zero in filaria-positive cases by 2022 (Figure 3) provides strong evidence of the success of national elimination strategies, including MDA, health education, and vector control efforts. Achieving zero cases by 2022 indicates the effective interruption of transmission, aligning with the global goal of eliminating LF as a public health problem. However, post-elimination surveillance is essential to ensure that transmission does not re-emerge.

The higher infection rate in males, 285 (64.62%) (Table 2), could be attributed to increased occupational or behavioural exposure to mosquito bites, particularly among men working outdoors or in environments conducive to vector breeding. These findings highlight the need for targeted health interventions and protective measures among adult males in endemic zones.

The age-stratified analysis (Table 3) showed that younger age groups (6-15 years) may indicate early exposure to infection before control programs achieved full effectiveness or gaps in coverage in the earlier phases of intervention. Additionally, this pattern suggests that younger individuals, due to their still-developing immune systems and increased environmental exposure, may be more susceptible to infection [13]. Adults are likely to have acquired partial immunity over time, resulting in lower parasitaemia levels. The findings suggest the importance of early detection and regular monitoring in school-aged populations, who can serve as a sentinel group for identifying any lingering transmission.

This retrospective study, conducted in the Nagpur district, assessed the long-term impact of MDA programmes, where a notable drop in MF prevalence was observed, from 4.91% in 2004 to 0% in 2024 (Table 5). This decline highlights the effectiveness of sustained MDA campaigns over two decades and reflects India's progress toward meeting the WHO's goal of eliminating LF as a public health problem. The sustained decline in MF rates across the Nagpur district is a direct outcome of systematically implemented MDA campaigns. Initially, these campaigns relied on the two-drug combination of diethylcarbamazine (DEC) and albendazole. However, a pivotal advancement occurred in January 2019, when Nagpur adopted the triple-drug regimen (ivermectin, DEC, and albendazole, known as IDA). This shift marked a significant intensification of the elimination strategy. Since the introduction of IDA in 2019, Nagpur has consistently recorded an MF rate of 0%, underscoring the superior efficacy of the triple-drug approach in halting parasite transmission. The success of this enhanced regimen illustrates the critical role of pharmacological innovation in accelerating LF elimination and aligns with the WHO's recommendation to use IDA in regions where traditional two-drug MDA has plateaued in impact.

Multiple studies further validate this trend. El-Setouhy et al. [14] documented a sharp decline in microfilaremia and antigenemia following multiple MDA rounds. Similarly, Coulibaly et al. [15] demonstrated a substantial decrease in the number of infective mosquito bites per person per year after successive MDA interventions. The current findings from Nagpur support this evidence base, confirming that both the longevity and optimization of MDA protocols, particularly the integration of IDA, are central to achieving and sustaining transmission interruption.

India's LF elimination efforts are aligned with WHO's global strategy and guided nationally by the NVBDCP. The NVBDCP has been central in operationalizing MDA campaigns and ensuring drug coverage above the critical 65% threshold needed to interrupt transmission [16,17]. The integration of vector control, health education, and drug distribution has been pivotal to success in several districts, including Nagpur.

Elimination progress has varied across states due to differing levels of healthcare infrastructure and community involvement. Southern states, such as Tamil Nadu and Kerala, achieved early reductions in LF due to better health systems and higher MDA compliance. In contrast, highly endemic states such as Bihar and Uttar Pradesh continue to face difficulties due to inadequate healthcare delivery and inconsistent programme implementation [16,17].

TAS has emerged as a critical tool in guiding the cessation of MDA programs and monitoring for resurgence. These surveys evaluate whether transmission has been interrupted and if the district meets the WHO criteria to halt MDA. Their implementation has strengthened post-MDA surveillance and minimized the risk of re-emergence by ensuring that only eligible districts stop drug administration [17,18].

While medical strategies have proven effective in reducing transmission, long-term elimination of LF requires addressing chronic cases and the associated social stigma. Continued surveillance, morbidity management, community education, and socio-economic rehabilitation are essential to consolidating elimination gains and preventing re-emergence. Emphasizing a people-centred, integrated approach will ensure that both clinical and social dimensions of LF are addressed sustainably.

Limitations

This study has several limitations that should be acknowledged. Firstly, the data relied exclusively on reported MF cases, which may not capture the true burden of infection in the community due to potential underreporting or limited access to diagnostic services in some areas. Secondly, as a retrospective analysis covering a span of two decades, there was insufficient clinical information available for many of the suspected cases, limiting the ability to correlate laboratory findings with clinical presentations. Additionally, there were gaps in surveillance during certain years, specifically 2016, 2020, and 2021, when surveys were not conducted. These missing data points may hinder the accurate interpretation of year-to-year trends and obscure the continuity of transmission patterns over time. Additionally, potential bias may have been introduced by changes in surveillance sites, particularly with the inclusion of two additional PHCs (Takalghat and Borkhedi) from 2021 onwards, which could have influenced case detection and comparability across years. Despite these limitations, the study provides valuable insights into the long-term epidemiological trends of LF in the region.

## Conclusions

This study highlights a significant public health achievement, marked by a decline in the MF rate from 4.91% in 2004 to 0% in 2024, underscoring the effectiveness of sustained MDA and robust community-based interventions in the Nagpur district. The balanced gender representation and consistent outreach reflect an inclusive and well-coordinated approach. This achievement not only demonstrates the power of persistent public health efforts but also offers a replicable model for LF elimination in other endemic regions. Maintaining this progress will require ongoing surveillance, community involvement, and adaptive strategies to ensure LF remains a disease of the past.
